# Tetrahydrocurcumin Outperforms Curcumin in Preventing Oxidative Stress-Induced Dysfunction in Tert-Butyl Hydroperoxide-Stimulated Cardiac Fibroblasts

**DOI:** 10.3390/ijms26135964

**Published:** 2025-06-21

**Authors:** Patrícia dos Santos Azeredo, Charity Fix, Laena Pernomian, Camilla F. Wenceslau, Gerardo G. Piroli, Cristina Pontes Vicente, Wayne E. Carver

**Affiliations:** 1 Department of Molecular and Morphofunctional Biology, State University of Campinas, Campinas 13083-865, SP, Brazil; p264504@dac.unicamp.br (P.d.S.A.); cvicente@unicamp.br (C.P.V.); 2 Department of Cell Biology and Anatomy, Cardiovascular Translational Research Center, School of Medicine, University of South Carolina, Columbia, SC 29209, USA; charity.fix@uscmed.sc.edu (C.F.); laena.pernomian@uscmed.sc.edu (L.P.); camilla.wenceslau@uscmed.sc.edu (C.F.W.); 3 Department of Pharmacology, Physiology & Neuroscience, School of Medicine, University of South Carolina, Columbia, SC 29209, USA; gerardo.piroli@uscmed.sc.edu

**Keywords:** oxidative stress, tetrahydrocurcumin, curcumin, apoptosis, *Tgfb1*, cardiac fibroblasts

## Abstract

Oxidative stress is a common feature of various pathological conditions, including tissue remodeling and dysfunction. Cardiac fibroblasts, which play a key role in maintaining extracellular matrix homeostasis, are sensitive to oxidative injury. Curcumin and tetrahydrocurcumin are plant-derived polyphenols with antioxidant properties, yet their relative efficacy in preventing oxidative stress–induced dysfunction in cardiac fibroblasts remains unclear. In this study, cardiac fibroblasts were treated with curcumin or tetrahydrocurcumin prior to exposure to tert-butyl hydroperoxide (t-BHP), a widely used inducer of oxidative stress. Cell viability, apoptosis, reactive oxygen species (ROS) production, and *Tgfb1* expression were assessed. Both curcuminoids significantly attenuated oxidative stress–induced cell death, decreased cell viability, and reduced *Tgfb1* expression. Notably, tetrahydrocurcumin demonstrated superior protective effects across most parameters. These findings suggest that both compounds help mitigate oxidative-stress–induced cellular dysfunction in cardiac fibroblasts and highlight tetrahydrocurcumin as a potentially more effective antioxidant. Further studies are needed to explore their role in the context of tissue remodeling and fibrotic progression.

## 1. Introduction

Cardiovascular diseases are the leading cause of death both in the United States and worldwide, accounting for 19.91 million deaths globally and 931,578 deaths in the United States in 2021. Additionally, these diseases incurred an estimated cost of USD 422.3 billion in the United States alone between 2019 and 2020 [1]. A major hallmark of many cardiovascular diseases is myocardial remodeling, often accompanied by cellular dysfunction and alterations in extracellular matrix (ECM) dynamics, which may contribute to pathological outcomes such as fibrosis and heart failure [2,3,4].

The heart is composed of a complex system of cellular and non-cellular components, including cardiac myocytes and fibroblasts embedded in a dense ECM. Cardiac fibroblasts play a central role in maintaining ECM homeostasis and responding to injury by regulating ECM synthesis and degradation. Under pathological stimuli, however, fibroblasts may adopt an activated phenotype and contribute to excessive ECM deposition, altered mechanical properties, and impaired electrical signaling—all of which are implicated in the progression of cardiac dysfunction [5,6,7,8,9,10]. Therefore, cardiac fibroblasts have received increased attention as potential therapeutic targets in heart disease [11,12,13].

Increased levels of reactive oxygen species (ROS) and oxidative stress are intimately associated with cellular dysfunction in fibroblasts [11,14]. Oxidative stress can result in widespread damage to macromolecules, including lipids, proteins, and nucleic acids, leading to systemic dysfunction and triggering cell death mechanisms like apoptosis and necrosis. Tert-butyl hydroperoxide (t-BHP) is an organic peroxide that can be metabolized by cells through cytochrome P450, producing peroxyl and alkoxyl radicals, or detoxified into tert-butanol. As a result, it rapidly oxidizes and depletes cellular glutathione, a tripeptide with an important antioxidant function [15]. Due to this property, t-BHP leads to cellular oxidative stress and is commonly used as an exogenous inducer of oxidative stress in both in vitro and in vivo laboratory research [16], including studies in fibroblasts [16,17,18,19]. In this context, compounds with antioxidant properties may help mitigate oxidative stress–induced dysfunction in cardiac fibroblasts, potentially contributing to preventive strategies in the early stages of cardiac remodeling.

Extracts from diverse plants are widely used in traditional medicine, and the World Health Organization estimates that over 80% of the global population has used plant-based compounds to prevent or treat various conditions [20]. One major advantage of plant-derived compounds is their ability to modulate multiple disease processes while generally exhibiting low toxicity and minimal side effects. *Curcuma longa*, a rhizomatous herbaceous plant native to India and Southeast Asia, has been used in traditional Indian and Chinese medicine for centuries. Its most prominent product, turmeric, is well known for its medicinal properties, which are largely attributed to a group of polyphenolic compounds known as curcuminoids. The main curcuminoids include curcumin, demethoxycurcumin, bisdemethoxycurcumin, and tetrahydrocurcumin, with curcumin representing approximately 75% of the curcuminoids found in commercial preparations [21].

Curcumin has been widely studied due to its abundance and reported biological effects in various models of inflammation, oxidative stress, and tissue remodeling [22,23]. Tetrahydrocurcumin, a major metabolite of curcumin, has demonstrated protective effects in experimental models of cardiovascular and metabolic diseases, including myocardial ischemia, chronic kidney disease, and diabetic cardiomyopathy [24,25]. These studies have shown its potential to attenuate myocardial remodeling, oxidative stress, and apoptosis. Moreover, tetrahydrocurcumin has been reported to possess greater antioxidant and anti-inflammatory activity than curcumin in several systems [26,27,28,29].

Despite growing interest in these compounds, direct comparisons of their relative efficacy in protecting cardiac fibroblasts from oxidative stress–induced dysfunction remain limited. Given the key role of fibroblasts in cardiac remodeling and their sensitivity to oxidative damage, the goal of the present study was to assess the relative ability of curcumin and tetrahydrocurcumin to prevent t-BHP–induced oxidative stress, cell death, and changes in gene expression associated with fibroblast dysfunction.

## 2. Results

### 2.1. Curcumin and Tetrahydrocurcumin Prevent t-BHP-Induced Reduction in Cell Viability

**Figure 1:**
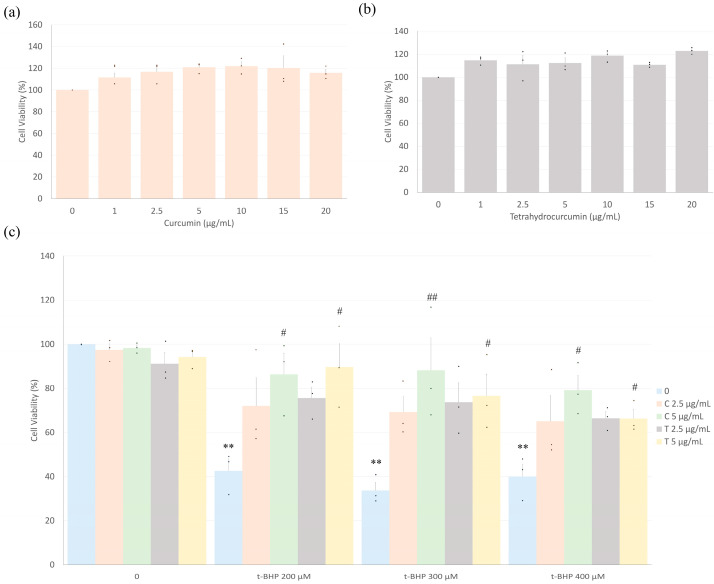
Curcumin and tetrahydrocurcumin prevent t-BHP-induced reduction in cardiac fibroblast viability. Fibroblasts were treated with different concentrations (2–20 µg/mL) of curcumin (**a**) or tetrahydrocurcumin (**b**) for 24 h, and cell viability was assessed using the methylthiazol tetrazolium (MTT) assay. (**c**) Cardiac fibroblast viability was evaluated after treatment with curcumin or tetrahydrocurcumin (2.5 and 5 μg/mL) for 24 h, followed by exposure to 0 (control), 200, 300, or 400 μM t-BHP for 1 h. Data are presented as mean ± S.E.M. (*n* = 3). Numbers above the bars indicate statistical comparisons: ** *p* < 0.01 vs. the untreated group (no t-BHP, curcumin, or tetrahydrocurcumin); ^#^ *p* < 0.05 vs. the corresponding dose of t-BHP alone; ^##^ *p* < 0.01 vs. the corresponding dose of t-BHP alone. One-way ANOVA followed by Tukey’s post hoc test (*p* < 0.05).

Cardiac fibroblasts (CFs) were treated with varying concentrations (0 to 20 µg/mL) of curcumin and tetrahydrocurcumin alone to assess the potential cytotoxicity of these compounds in isolated rat cardiac fibroblasts. The results indicated that individually, none of the concentrations tested of either compound demonstrated a negative impact on cell viability (Figure 1a,b). In fact, there was a trend towards improved cell viability with both compounds compared to the untreated control (absence of either compound). Based on these data, further experiments were carried out using 2.5 and 5 µg/mL concentrations of each compound.

Cardiac fibroblasts were treated with varying concentrations of t-BHP (200, 300, or 400 µM) with or without curcumin or tetrahydrocurcumin pretreatment, and cell viability was assessed. All concentrations of t-BHP tested significantly decreased the viability of CFs (blue bars in Figure 1c). At 2.5 µg/mL, both curcuminoids showed an increase in cell viability compared to the group treated with t-BHP alone, although this difference did not reach statistical significance. Pretreatment with 2.5 µg/mL curcumin or tetrahydrocurcumin also did not differ significantly from the untreated control, suggesting a trend toward attenuation of t-BHP-induced cytotoxicity. Pretreatment of cells with curcumin or tetrahydrocurcumin significantly attenuated the effects of t-BHP on CF viability at 5 µg/mL.

### 2.2. Pretreatment with Curcumin and Tetrahydrocurcumin Prevents t-BHP-Induced Apoptosis

**Figure 2:**
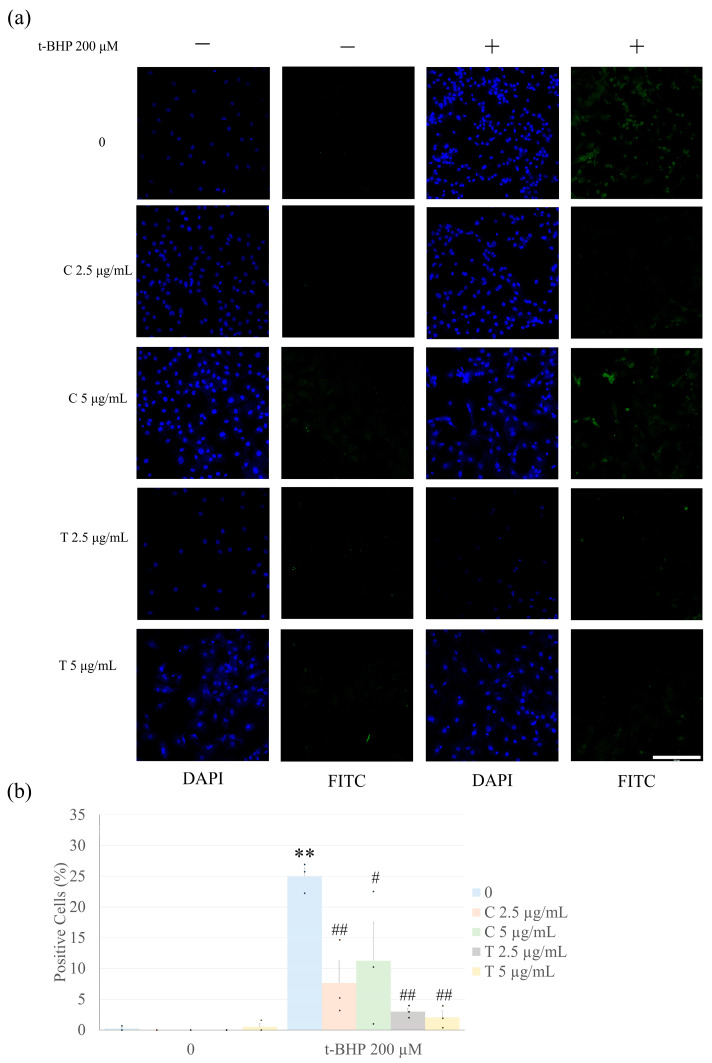
Curcumin and tetrahydrocurcumin prevent t-BHP-induced CF apoptosis. CFs were pretreated with curcumin or tetrahydrocurcumin (2.5 or 5 µg/mL) for 24 h, followed by exposure to 200 μM t-BHP for 1 h, and analyzed by the TUNEL assay. Data is shown as mean ± S.E.M. (*n* = 3). Numbers above the bars indicate statistical comparisons: ** *p* < 0.01 vs. 0 (no t-BHP, curcumin, or tetrahydrocurcumin). ^#^ *p* < 0.05 vs. 200 μM t-BHP alone; ^##^ *p* < 0.01 vs. 200 μM t-BHP alone. One-way ANOVA, followed by Tukey’s post hoc test (*p* < 0.05). C = Curcumin; T = Tetrahydrocurcumin. Representative fluorescent images for 4’,6-diamidino-2-phenylindole (DAPI) (blue) and TUNEL (green) in (**a**). The bar graph shows TUNEL-positive cells (%) in (**b**). Scale bar = 200 μm.

Due to the effect of t-BHP on cell viability at 200 µM and the lack of a difference between 200, 300, and 400 µM, analysis of apoptosis was performed with 200 µM t-BHP. Treatment of CFs for 1 h with 200 µM t-BHP significantly increased apoptosis based upon the percentage of cells that were positive for Terminal deoxynucleotidyl transferase deoxyuridine triphosphate nick end labeling (TUNEL) staining (Figure 2). However, pretreatment with all concentrations of tetrahydrocurcumin and curcumin tested significantly reduced t-BHP-induced fibroblast apoptosis. Notably, tetrahydrocurcumin demonstrated greater effectiveness, as both 2.5 µg/mL and 5 µg/mL concentrations significantly reduced apoptosis (*p* < 0.01). In contrast, curcumin at 5 µg/mL was less effective than at 2.5 µg/mL and also less effective than both concentrations of tetrahydrocurcumin, supporting the superior antiapoptotic activity of tetrahydrocurcumin in this model.

### 2.3. Curcumin and Tetrahydrocurcumin Pretreatment Prevent Intracellular ROS Production

**Figure 3:**
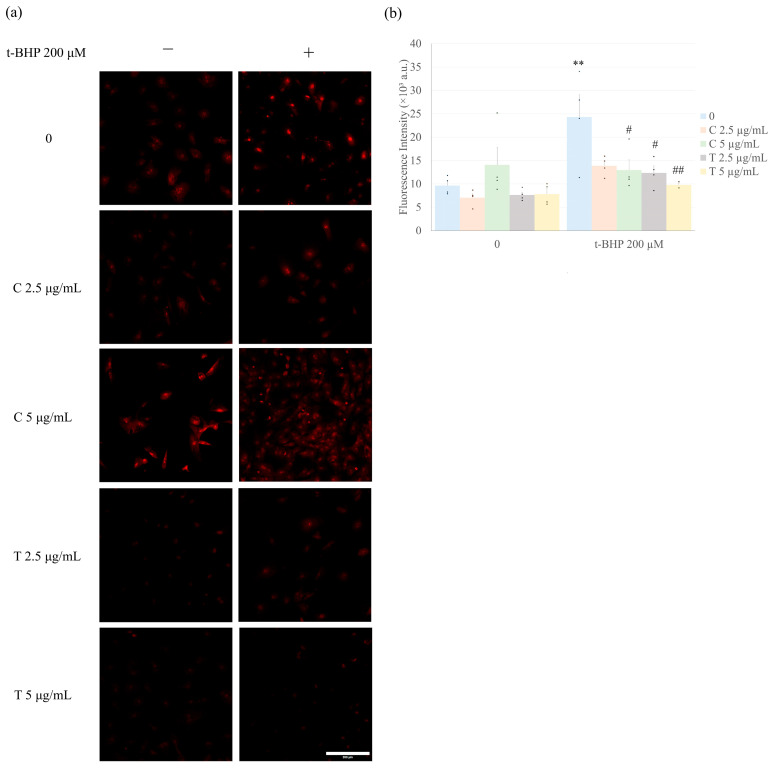
Antioxidant effect induced by curcumin and tetrahydrocurcumin in t-BHP-treated CF. CFs were treated with different concentrations (2.5 or 5 µg/mL) of curcumin or tetrahydrocurcumin for 23 h, followed by exposure to 200 μM t-BHP for 1 h in the continued presence of curcuminoids. ROS production was evaluated by Dihydroethidium (DHE) staining. Data are shown as mean ± S.E.M. (*n* = 4). Numbers above the bars indicate statistical comparisons: ** *p* < 0.01 vs. 0 (no t-BHP, curcumin, nor tetrahydrocurcumin). ^#^ *p* < 0.05 vs. 200 μM t-BHP alone; ^##^ *p* < 0.01 vs. 200 μM t-BHP alone. One-way ANOVA, followed by Tukey’s post hoc test (*p* < 0.05). C = Curcumin; T = Tetrahydrocurcumin. Representative images for DHE (red) in (**a**), and the graph shows quantitative analysis of DHE fluorescence intensity in (**b**). Scale bar = 200 μm. Fluorescence intensity is expressed in arbitrary units (a.u.).

As shown in Figure 3, the treatment of CFs with 200 µM of t-BHP alone for 1 h significantly increased ROS production in the cells as detected by dihydroethidium (DHE). Treatment of CFs with curcumin or tetrahydrocurcumin (2.5 and 5 µg/mL) alone did not affect ROS levels. However, pretreatment with curcumin or tetrahydrocurcumin effectively and similarly prevented the t-BHP-induced increase in ROS production, with the exception of curcumin at 2.5 µg/mL, which did not significantly prevent the ROS increase (Figure 3).

### 2.4. Antioxidant Enzyme Expression

**Figure 4:**
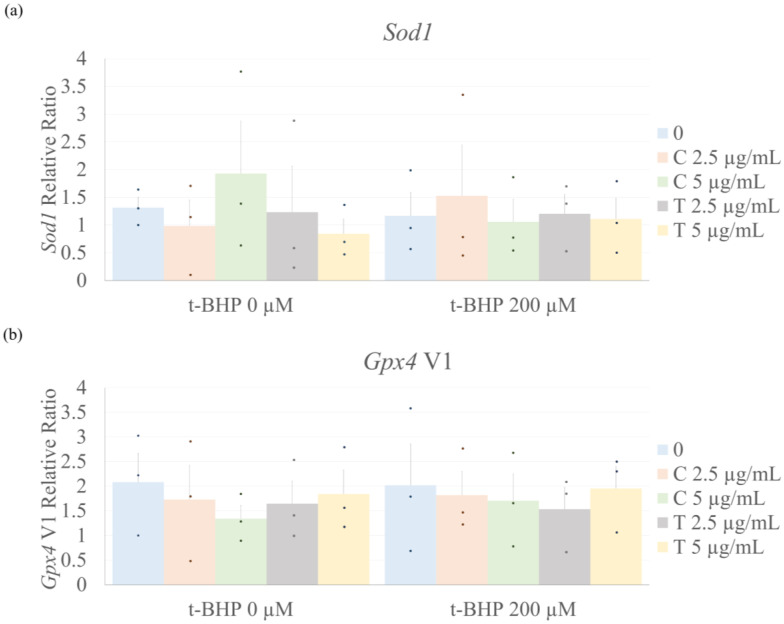
Curcumin or tetrahydrocurcumin does not change antioxidant enzyme gene expression in CF. Effect of curcuminoids on *Sod1* and *Gpx4* V1 mRNA expression in CF measured by qRT-PCR. Data are shown as mean ± SEM (*n* = 3) and normalized by the housekeeping gene acidic ribosomal binding protein (ARBP). One-way ANOVA, followed by Tukey’s post hoc test (*p* < 0.05). C = Curcumin; T = Tetrahydrocurcumin. (**a**) SOD1 relative expression, (**b**) GPx4V1 relative expression.

Due to changes identified in levels of ROS following the treatment of CF with t-BHP and the curcuminoids, we then performed quantitative reverse transcriptase-polymerase chain reaction (qRT-PCR) to assess the relative mRNA expression of representative antioxidant enzymes. Neither t-BHP nor the curcuminoids, alone or in combination with t-BHP, induced significant changes in the gene expression of superoxide dismutase (*Sod1*) 1 or glutathione peroxidase 4 (*Gpx4*) v1 (Figure 4).

### 2.5. Effects of Treatment on Collagen Gel Contraction

**Figure 5:**
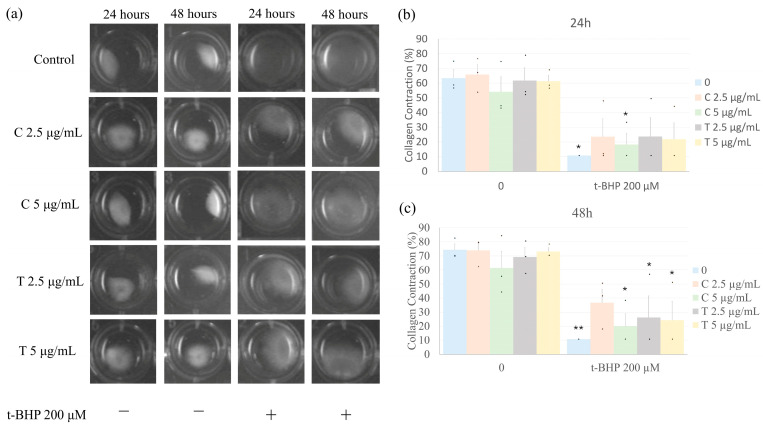
Curcumin and tetrahydrocurcumin do not induce collagen gel contraction. CFs were pretreated with different concentrations (2.5 or 5 µg/mL) of curcumin or tetrahydrocurcumin for 24 h, followed by exposure to 200 μM t-BHP for 1 h (prior to the end of the 24 h), and the collagen gel contraction assay was evaluated after 24 or 48 h. Data are shown as mean ± SEM (*n* = 3). Numbers above the bars indicate statistical comparisons: * *p* < 0.01 vs. 0 (no t-BHP, curcumin, nor tetrahydrocurcumin); ** *p* < 0.01 vs. 0. One-way ANOVA, followed by Tukey’s post hoc test (*p* < 0.05). C = Curcumin; T = Tetrahydrocurcumin. Representative images of collagen gel contraction (**a**) and bar graphs (%) after 24 (**b**) or 48 h (**c**).

Culture of fibroblasts in three-dimensional collagen hydrogels results in the contraction of the hydrogels via interactions of the cells with the collagenous matrix. Treatment of collagen hydrogels containing CF with t-BHP alone for 24 or 48 h resulted in reduced collagen contraction compared to collagen gels cultured without t-BHP (blue bars in Figure 5). Pretreatment with either dose of curcumin or tetrahydrocurcumin, followed by t-BHP treatment, had no significant effect on the inhibition of collagen hydrogel contraction by t-BHP. After 24 h, all of the combined groups (t-BHP + curcuminoids) demonstrated a trend towards inhibited contraction; however, only the group treated with 5 µg/mL curcumin was significantly different. By 48 h, contractions in all of the treatment groups were significantly inhibited compared to the control group, except for the group pretreated with 2.5 µg/mL curcumin.

### 2.6. Biomarkers of Fibrosis

**Figure 6:**
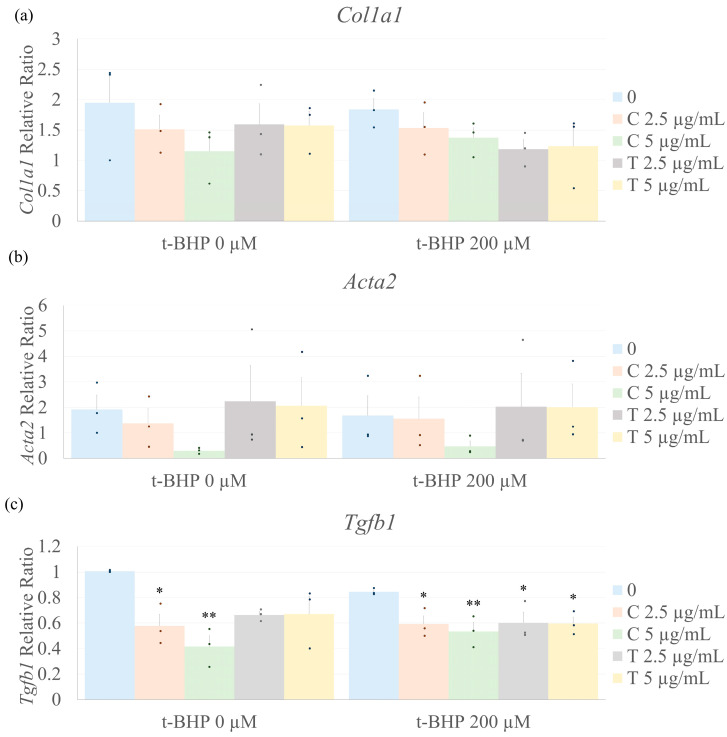
Curcumin and tetrahydrocurcumin decrease *Tgfb1* gene expression in cardiac fibroblasts. Effect of curcuminoids on *Colla1*, *Acta2* (**b**), and *Tgfb1* (**c**) mRNA expression in cardiac fibroblasts. Data are shown as mean ± SEM (*n* = 3) and normalized by the housekeeping gene ARBP. Numbers above the bars indicate statistical comparisons: * *p* < 0.05 vs. 0 (no t-BHP, curcumin, or tetrahydrocurcumin). ** *p* < 0.01 vs. 0 (no t-BHP, curcumin, nor tetrahydrocurcumin). One-way ANOVA, followed by Tukey’s post hoc test (*p* < 0.05). C = Curcumin; T = Tetrahydrocurcumin. (**a**) *Colla1*, (**b**) *Acta2* relative expression, (**c**) *Tgfb1* relative expression.

Fibrosis is characterized by the activation of fibroblasts and enhanced expression of ECM components and pro-fibrotic cytokines. Treatment of CF with t-BHP for 1 h had no effect on collagen α1(I), α-smooth muscle actin (α-SMA), or TGF-β1 mRNA expression (Figure 6). Neither t-BHP, curcumin, nor tetrahydrocurcumin alone or in combination affected Col1a1 nor Acta2 mRNA levels. Interestingly, treatment with curcumin alone and in combination with t-BHP reduced *Tgfb1* mRNA expression. Additionally, tetrahydrocurcumin at 2.5 and 5.0 µg/mL in the presence of t-BHP decreased *Tgfb1* expression compared to untreated cells (no t-BHP nor tetrahydrocurcumin). In the absence of t-BHP, tetrahydrocurcumin reduced *Tgfb1* mRNA expression; however, this was not statistically significant.

## 3. Discussion

Cardiac fibroblasts are responsible for producing the majority of the extracellular matrix (ECM) and play a central role in maintaining tissue structure and response to stress. Their dysfunction is closely associated with detrimental remodeling processes in the heart, which may contribute to impaired mechanical and electrical function [4,30]. Because of their key role in cardiac homeostasis, fibroblasts have become a focus of interest in the development of new therapeutic approaches. Curcumin has been widely studied and has shown the potential to modulate oxidative stress and cellular dysfunction in cardiovascular cell types, as well as influence markers associated with remodeling and fibrosis [22,23,31]. Notably, tetrahydrocurcumin, a major metabolite of curcumin, has also demonstrated protective effects in the cardiovascular system, including attenuation of oxidative damage and dysfunction in several experimental models [24,25]. Some evidence suggests that tetrahydrocurcumin may exert superior effects compared to curcumin in certain contexts [32]. However, its direct effects on cardiac fibroblasts under oxidative stress remain underexplored, and further preclinical studies are needed to clarify its mechanisms of action in preventing fibroblast dysfunction and associated pathological remodeling.

Oxidative stress is strongly associated with cellular dysfunction, often marked by increased production of reactive oxygen species (ROS) and elevated levels of apoptosis [33]. Previous studies [19,34,35] have shown that t-BHP induces apoptosis and reduces cell viability in fibroblasts in a concentration-dependent manner. We observed that 200, 300, and 400 µM t-BHP reduced CF viability, with 200 µM also increasing apoptotic cell numbers. Oxidative stress–induced dysfunction can also stimulate the release of stress-responsive cytokines such as TGF-β [36], which are involved in multiple cellular pathways, including inflammatory and remodeling responses [37]. Both curcuminoids were effective in preventing t-BHP-induced reductions in cell viability and apoptosis. Notably, tetrahydrocurcumin exhibited superior protective effects compared to curcumin in this context, in agreement with previous studies reporting its enhanced bioactivity [32].

TGF-β is a pleiotropic cytokine involved in a variety of cellular processes, including responses to oxidative stress, inflammation, and tissue remodeling [37,38]. Its expression is frequently upregulated under oxidative stress, although this response may vary depending on stimulus duration, cell type, and context [39]. In this study, brief exposure (1 h) of cardiac fibroblasts to t-BHP did not alter *Tgfb1* mRNA expression. However, pretreatment with curcumin or tetrahydrocurcumin for 24 h significantly reduced *Tgfb1* mRNA levels, regardless of t-BHP exposure. These findings are in line with prior studies showing that curcumin can downregulate *Tgfb* expression [40] and interfere with its signaling pathway [41]. While few studies have directly examined tetrahydrocurcumin’s effect on *Tgfb*, one report noted increased *Tgfb* levels in the frontal cortex of mice under chronic stress following tetrahydrocurcumin treatment [42], suggesting its effects may be highly context- or cell-specific. Although t-BHP has been used in other models to mimic fibrotic conditions, such as in renal epithelial cells, where it increased collagen and fibronectin expression [43], in our study, it did not alter the expression of fibrotic markers, including *Tgfb1*, *Acta2*, and *Col1a1*. This lack of effect may be due to the relatively short exposure time in our model, which may have been sufficient to trigger oxidative stress and early cellular dysfunction, but not long enough to induce downstream changes in gene expression related to remodeling or matrix production.

Oxidative stress can trigger a number of changes in fibroblast phenotype and behavior, including the conversion to myofibroblasts that have enhanced contractile properties and elevated expression of *Acta2*, the gene encoding alpha-smooth muscle actin [44]. A commonly used indirect method to assess this contractile phenotype is the ability of fibroblasts to contract 3D collagen hydrogels in vitro. In the present study, the control group exhibited pronounced collagen hydrogel contraction at both 24 and 48 h, likely due to the spontaneous differentiation of fibroblasts into myofibroblasts in response to growth factors in the serum-containing medium and the mechanical forces of the 3D collagen environment. In contrast, treatment with t-BHP substantially inhibited collagen hydrogel contraction, which we attribute, at least in part, to t-BHP-induced cell death, as supported by viability and apoptosis assays. Pretreatment with curcumin (2.5 µg/mL) or tetrahydrocurcumin (2.5 and 5 µg/mL) partially mitigated this effect at 24 h. That is, contraction of collagen gels in the presence of the curcuminoids and t-BHP was not significantly different from t-BHP alone nor from untreated controls. The mechanism whereby curcumin and tetrahydrocurcumin counter the inhibitory effects of t-BHP on collagen hydrogel contraction by CFs is not known. Interestingly, these results are in alignment with recent studies illustrating that curcumin alone or in combination with an engineered dermal matrix hydrogel accelerated wound contraction in a diabetic mouse model [45]. Further studies will be required to evaluate the cellular and molecular mechanisms of this response.

ROS play a crucial role in cellular dysfunction, particularly when there is an imbalance between the cell’s antioxidant and oxidative potential, a condition known as oxidative stress. This increase in ROS can lead to membrane oxidation, DNA damage, and even cell death, further amplifying the oxidative response [46]. Curcumin has been shown to mitigate oxidative stress through various mechanisms, including directly binding with ROS [47] and enhancing the activity of antioxidant enzymes such as catalase [48]. However, the effects of tetrahydrocurcumin on ROS production have not been fully elucidated. These findings highlight a distinct difference in the antioxidant behavior of curcumin and tetrahydrocurcumin. Although both curcumin and tetrahydrocurcumin were evaluated at two concentrations, their antioxidant efficacy differed. Curcumin showed a protective effect only at the higher dose, while tetrahydrocurcumin was effective at both concentrations, with a more pronounced effect at the higher dose. This suggests that tetrahydrocurcumin may have a more stable redox-modulating profile, making it a potentially more reliable agent for preventing oxidative stress in this cellular context. The expression of antioxidant enzymes, such as *Sod1* [49] and *Gpx4* [50], typically increases in response to oxidative stress to mitigate ROS accumulation. In this study, however, the mRNA levels of *Sod1* and *Gpx4* did not change in response to t-BHP treatment, with or without curcuminoids. This indicates that the decrease in ROS production may be due to other mechanisms, possibly involving post-translational modifications, such as increased enzyme activity rather than transcriptional modulation, though these aspects were not assessed in this study.

## 4. Materials and Methods

### 4.1. Heart Fibroblasts Extraction and Cell Culture

CFs were isolated from 8-to-10-week-old male Sprague Dawley rats (Harlan Laboratories) following Stewart et al. [51]. After euthanasia by cervical dislocation under isoflurane anesthesia, hearts were excised, minced, and digested with Liberase (Roche Life Sciences) at 37 °C. Fibroblasts were purified through differential adhesion and cultured in DMEM with 10% FBS and 1% penicillin-streptomycin. Cells were maintained at 37 °C with 5% CO₂, passaged at 70–80% confluence, and only used up to passage 5 to preserve phenotype consistency. The purity of cardiac fibroblast cultures was routinely evaluated by immunocytochemical staining (Supplementary Figure S1) for markers of fibroblasts (vimentin and discoidin domain receptor 2) and other cardiac cell types including smooth muscle cells (SM22a), endothelial cells (CD31 and VE-cadherin), and macrophages (galectin 3 and CD68).

### 4.2. Fibroblast Treatment

Before the treatment phase commenced, the culture medium was switched to DMEM containing 1.5% fetal bovine serum (low-serum medium) for 24 h. The purpose of culturing in low serum was to minimize spontaneous fibroblast activation and conversion to a myofibroblast phenotype in response to serum components. Fibroblasts were grown to approximately 80% confluence and then pretreated with either vehicle alone (0.1% dimethylsulfoxide, DMSO), curcumin (2.5 or 5 µg/mL), or tetrahydrocurcumin (2.5 or 5 µg/mL) for 23 h prior to the addition of different concentrations of t-BHP. Following the 23 h pretreatment with curcumin or tetrahydrocurcumin, t-BHP was added to the cells while the curcuminoids remained present, and the cells were incubated for an additional 1 h. Cells treated only with vehicle served as negative controls.

### 4.3. Cell Viability Assay

Cell viability was evaluated using the MTT assay (Invitrogen, REF: V13154). CFs were seeded at a density of 5 × 10^3^ cells per well in a 96-well plate. After treatment as described above, MTT was added to each well at a concentration of 5 µg/mL and incubated for 1 h. The solution was then removed, and DMSO was added to dissolve the formazan crystals. Absorbance was measured at 490 nm using a microplate reader.

### 4.4. Oxidative Stress

To assess intracellular levels of ROS, dihydroethidium (DHE) (Thermo Fisher Scientific, Waltham, MA, USA; REF D11347) was used. Coverslips were placed at the bottom of 24-well plates and coated with 10 µg/mL collagen for 1 h prior to the experiment. CFs were plated onto coverslips at a concentration of 50,000 cells per well and treated with t-BHP with or without curcumin or tetrahydrocurcumin pretreatment as described above. Once the treatment period concluded, the wells were washed with Hanks’ solution with Mg^2+^ and Ca^2+^ (HBSS solution), and 10 µM DHE was added to each well, followed by a 30 min incubation at 37 °C in the dark. Subsequently, the coverslips with the cells were transferred to glass slides, mounted with Fluormount-GTM (Thermo Fisher Scientific—REF 00-4958-02), and immediately imaged using a confocal microscope (Leica Stellaris 5 LIAchroic Confocal System). The fluorescence intensity of intracellular DHE was measured using ImageJ (version 1.54f) by quantifying the nuclear fluorescence of at least 10 individual cell nuclei per group. This analysis was performed in 4 independent experiments (*n* = 4). The brightness of all images presented in the results was uniformly increased by 20% to improve visualization. This adjustment was applied equally to all images, and no other image processing was performed.

### 4.5. Apoptosis

For the analysis of apoptosis using TUNEL, the In Situ Cell Death Detection Kit (Fluorescein) was utilized (Sigma, St. Louis, MO, USA, catalog number 11684795910). Fibroblasts (50,000 per well) were plated on coverslips coated with 10 µg/mL of collagen in a 24-well plate, treated with t-BHP, curcumin, and tetrahydrocurcumin as described above, and stained according to the manufacturer’s protocol. Cells were also stained with 1 μg/mL of DAPI for 1 h to visualize all nuclei. Fluorescent images were captured using a confocal microscope (Leica Stellaris 5 LIAchroic Confocal System). The total number of apoptotic cells was quantified by counting the positively stained nuclei with TUNEL and reported as a percentage of all cells (determined by DAPI staining). The brightness of all images presented in the results was adjusted uniformly to improve visualization: DAPI images were increased by 70% and FITC images by 50%. These adjustments were applied equally across all samples, and no other image processing was performed.

### 4.6. Collagen Gel Contraction

Fibroblasts, when cultured in 3-dimensional collagen hydrogels, will interact with and contract the collagenous matrix [52]. This is particularly true if the fibroblasts are stimulated to produce myofibroblasts. The extent of gel contraction correlates with the number and activity of myofibroblasts present [53].

CF cells were plated in 100 mm plates and pretreated with curcumin and tetrahydrocurcumin, followed by treatment with t-BHP as described above. Following this, fibroblasts were trypsinized and resuspended in the same medium (including the corresponding doses of t-BHP, curcumin, and tetrahydrocurcumin). To create three-dimensional collagen hydrogels, fibroblasts were mixed with bovine collagen type I (PureCol; Advanced BioMatrix, Inc., San Diego, CA, USA) at a concentration of 50,000 cells per milliliter of collagen (1.2 mg/mL). This mixture was added to 24-well plates precoated with bovine serum albumin to prevent adhesion of collagen hydrogels to the culture plastic. Collagen hydrogels containing cells were allowed to polymerize at 37 °C for one hour. Once polymerized, the hydrogels containing CFs were detached from the plastic wells, and culture continued. After 24 and 48 h, the perimeter of the collagen gels was measured, and data were presented as the final perimeter of the hydrogels relative to their initial size. For better visualization, individual wells were cropped from the original gel image to create a labeled mosaic. The original, uncropped gel image is provided in the Supplementary Material. The wells corresponding to each experimental condition are indicated in the annotated version of the original image.

### 4.7. Quantitative Reverse Transcriptase-Polymerase Chain Reaction (qRT-PCR)

CFs were cultured and treated as described above. Following treatment, the cells were washed with phosphate-buffered saline and extracted using Trizol reagent (Invitrogen, Carlsbad, CA, USA). The RNA was precipitated, resuspended in nuclease-free water, and purified with a RNeasy Mini Kit (Qiagen, Valencia, CA, USA). The RNA concentration was determined using a spectrophotometer. cDNA synthesis was performed with 2 μg of RNA using the iScript cDNA kit (Bio-Rad, Hercules, CA, USA). qRT-PCR was carried out using specific primers (Integrated DNA Technologies—IDT) designed for fibrosis markers and oxidative stress (Table 1). Reactions were carried out in SYBR Green Supermix (Bio-Rad), and the relative expression of target mRNAs was normalized to acidic ribosomal binding protein (ARBP). The relative expression analysis was conducted using the 2^(−ΔΔCt)^ method, as described by Livak and Schmittgen [54].

### 4.8. Statistical Analysis

All data analysis was performed blinded to the treatment condition. All statistical analyses were performed using GraphPad Prism 8.4.3 (GraphPad Software Inc., La Jolla, CA, USA). Data are presented as mean ± standard error of the mean (S.E.M.), and statistical significance was set at *p* < 0.05. The One-way analysis of variance (ANOVA), followed by Tukey post hoc, was used to identify interaction factors and compare 3 or more groups, when data followed a normal distribution, and was analyzed by parametric tests. The sample size (*n*) indicated per experiment is the number of independent samples used.

## 5. Conclusions

There is growing interest in the use of plant-derived compounds to protect against oxidative stress–related cellular dysfunction. In this study, both curcumin and tetrahydrocurcumin effectively attenuated t-BHP–induced oxidative stress, reduced cell death, and decreased *Tgfb1* expression in cardiac fibroblasts. Notably, tetrahydrocurcumin demonstrated greater efficacy across most parameters. These findings suggest that curcuminoids, particularly tetrahydrocurcumin, may offer protective benefits against oxidative damage in cardiac fibroblasts. Future studies are needed to investigate their potential role in modulating fibroblast behavior in the broader context of tissue remodeling and disease progression.

## Figures and Tables

**Table 1:** Sequences of primers (rat) used for qRT-PCR.

Primer	Sequence
Collagen type I alpha 1 chain (*Col1a1*)	Forward: 5’-GCGAAGGCAACAGTCGATTC-3’
	Reverse: 5’CCCAAGTTCCGGTGTGACTC-3’
Alpha-smooth muscle actin (*Acta2*)	Forward: 5’-GGAGTGATGGTTGGAATGG-3’
	Reverse: 5’-ATGATGCCGTGTTCTATCG-3’
Transforming growth factor beta 1 (*Tgfb1*)	Forward: 5’TGCCCTCTACAACCAACACA-3’
	Reverse: 5’GTTGGACAACTGCTCCACCT-3’
Superoxide dismutase 1 (*Sod1*)	Forward: 5’-GGTGTGGCCAATGTGTCCATTGAA-3’
	Reverse: 5’-CGGCTTCCAGCATTTCCAGTCTTT-3’
Glutathione peroxidase 4 (*Gpx4*)	Forward: 5’-AGGCAGGAGCCAGGAAGTAATCAA-3’
	Reverse: 5’-CCTTGGGCTGGACTTTCATCCATT-3’
Acidic ribosomal phosphoprotein P0 (*Arbp*)	Forward: 5’-TAGAGGGTGTCCGCAATG-3’
	Reverse: 5’-GAAGGTGTAGTCAGTCTCC-3’

## Data Availability

The data that support the findings of this study are available from the corresponding author upon reasonable request.

## Supplementary Materials

The following supporting information can be downloaded at: https://www.mdpi.com/article/10.3390/ijms26135964/s1.

## Abbreviations

The following abbreviations are used in this manuscript:

t-BHP	Tert-butyl hydroperoxide
ROS	Reactive oxygen species
Tgfb1	Transforming growth factor beta
ECM	Extracellular matrix
CF	Cardiac fibroblasts
DMEM	Dulbecco’s modified Eagle’s medium
MTT	Methylthiazol tetrazolium
DHE	Dihydroethidium
TUNEL	Terminal deoxynucleotidyl transferase deoxyuridine triphosphate nick end labeling
DAPI	4’,6-diamidino-2-phenylindole
qRT-PCR	Quantitative reverse transcriptase-polymerase chain reaction
ARBP	Acidic ribosomal binding protein
SOD	Superoxide dismutase
GPx4	Glutathione peroxidase 4
α-SMA	α-smooth muscle actin
ANOVA	Analysis of variance
S.E.M	Standard error of the mean
