# Supplementary material for: Tetrahydrocurcumin Outperforms Curcumin in Preventing Oxidative Stress-Induced Dysfunction in Tert-Butyl Hydroperoxide-Stimulated Cardiac Fibroblasts
Source: Int J Mol Sci. 2025 Jun 21;26(13):5964. doi: 10.3390/ijms26135964 (PMC12250096; doi:10.3390/ijms26135964)
Supplement: Supplementary file 1 [file ijms-26-05964-s001.zip › ijms-3532234-supplementary.pdf]

Supplementary Materials

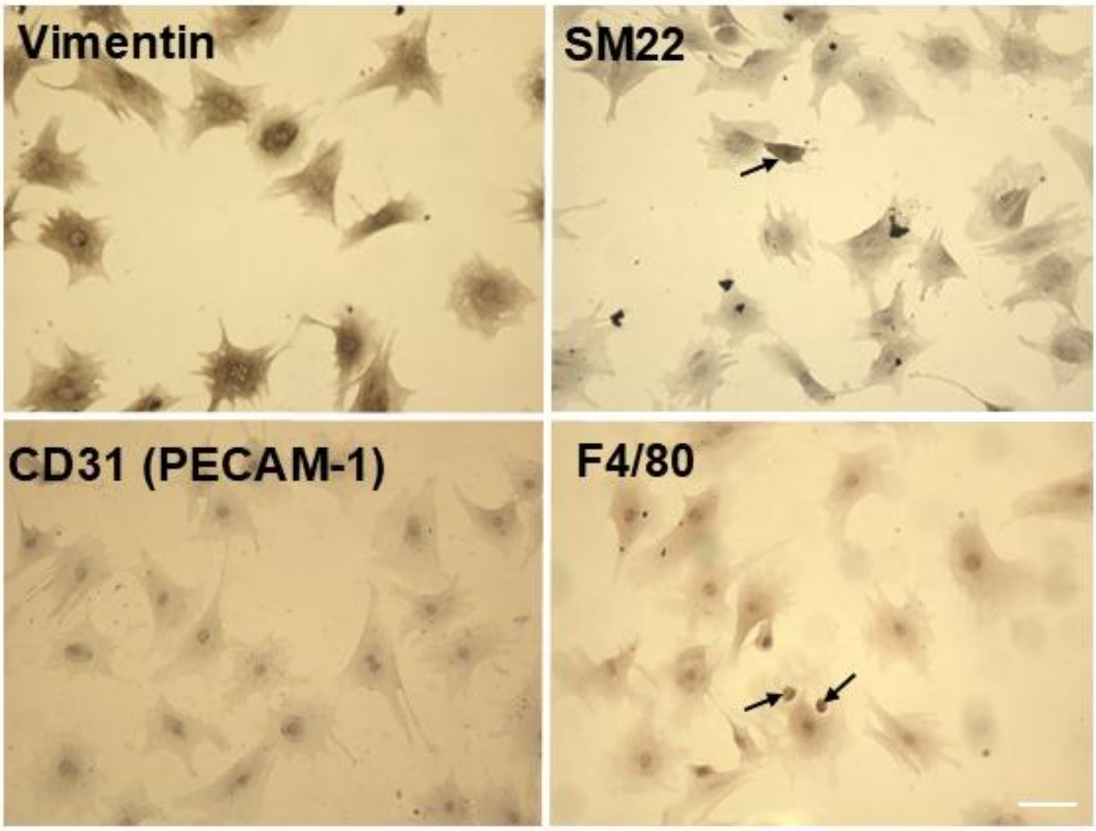

Figure S1. Cardiac fibroblasts were immunocytochemically stained at the second passage with antibodies against vimentin, SM22 alpha (smooth muscle cells), CD31 (endothelial cells) or F4/80 (macrophages) and horse radish peroxidase-conjugated secondary antibodies. Cells were counter-stained with hematoxylin to demonstrate nuclei. Greater than 95% of the cells typically stain positively for vimentin, less than 1% for CD31 and 1-2% for SM22 alpha and F4/80. The arrows indicate cells positively stained for SM22 alpha and F4/80.

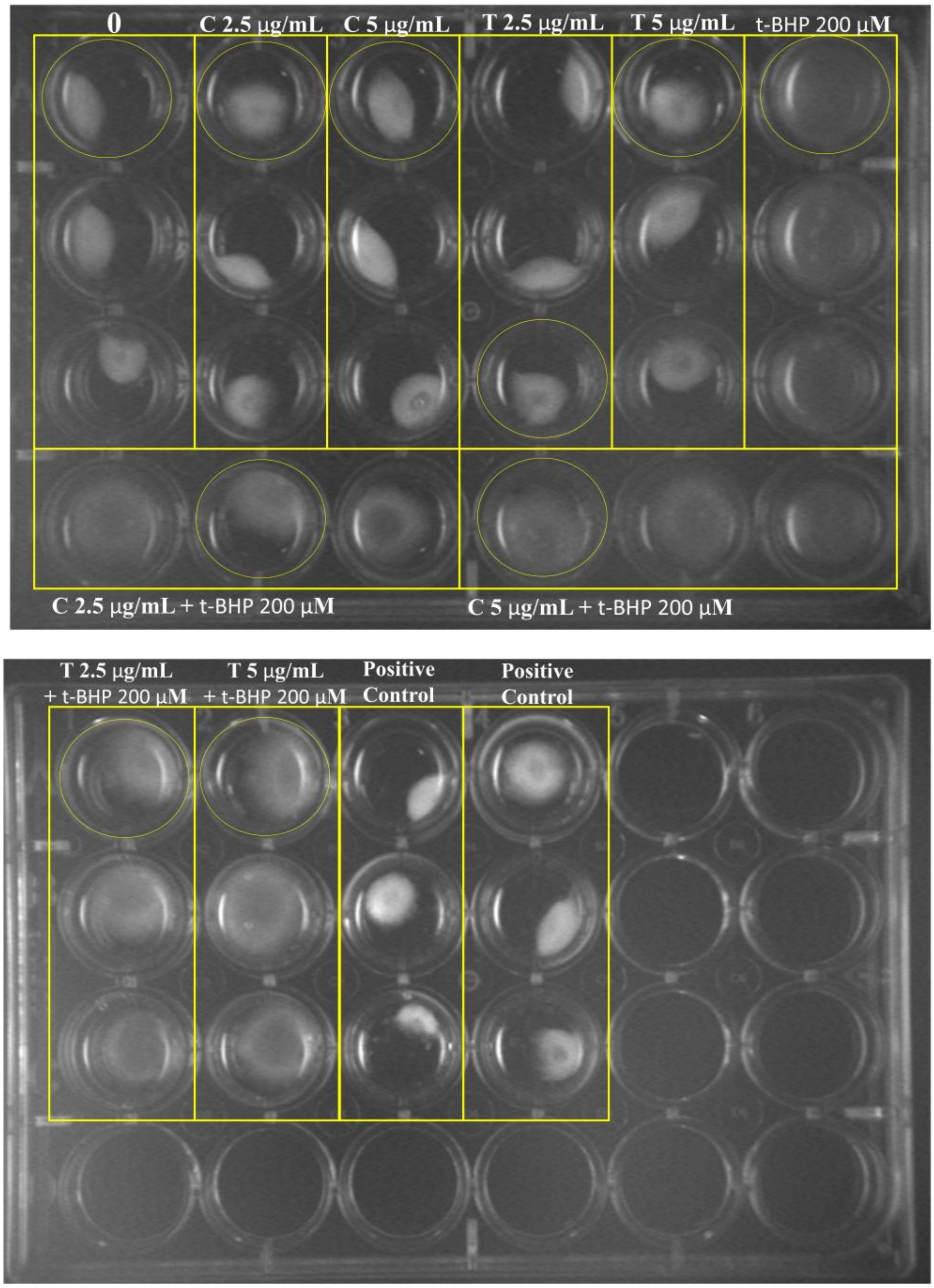

Figure S2. Original, uncropped gel images from the collagen gel contraction assay, taken after the 24-hour period. The wells used to create the mosaic in Figure 5 of the main article are highlighted in yellow.

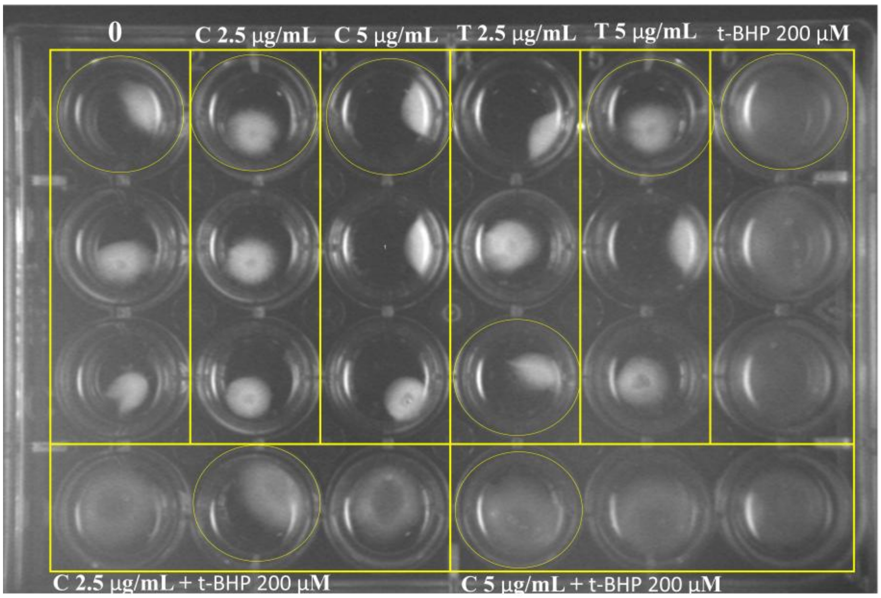

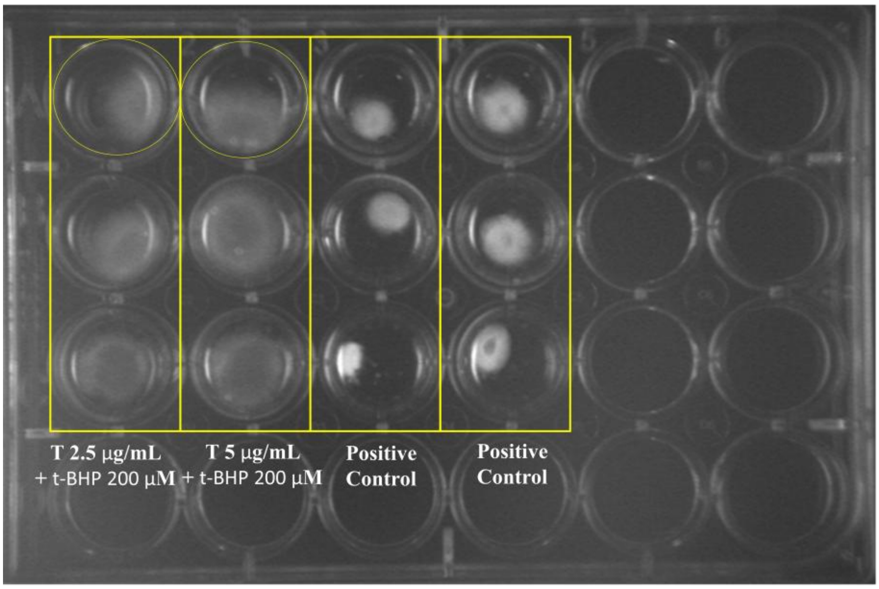

Figure S3. Original, uncropped gel images from the collagen gel contraction assay, taken after the 48-hour period. The wells used to create the mosaic in Figure 5 of the main article are highlighted in yellow.

Treatment Group	n (independent experiments)	Cells analyzed per experiment (n)	ROS intensity per cell nucleus (mean ± SE)	p-value (vs. 0)
0	4	10	9.65 ± 0.95	-
C 2.5 µg/mL	4	10	7.04 ± 0.85	0.9973
C 5 µg/mL	4	10	14.05 ± 3.75	0.9161
T 2.5 µg/mL	4	10	7.62 ± 0.61	0.9996
T 5 µg/mL	4	10	7.82 ± 1.11	0.9998
t-BHP 200 uM	4	10	24.32 ± 4.79	0.002
C 2.5 µg/mL + tBHP 200 uM	4	10	13.85 ± 1.03	0.935
C 5 µg/mL + tBHP 200 uM	4	10	12.94 ± 2.26	0.9859
T 2.5 µg/mL + tBHP 200 uM	4	10	12.34 ± 1.51	0.9966
T 5 µg/mL + tBHP 200 uM	4	10	9.80 ± 0.39	>0,9999
**Total/group**		**40**		

Table S1. Quantification of intracellular reactive oxygen species (ROS) levels in CF cells treated with t-BHP and pre-treated with curcumin or tetrahydrocurcumin, as assessed by dihydroethidium (DHE) staining. For each group, 10 individual cell nuclei were measured to quantify nuclear fluorescence intensity. The table shows the number of independent experiments (n), the number of nuclei measured per group, the mean fluorescence intensity (in arbitrary units) and the standard error (SE) for each group. Data represent the average of four independent experiments (n = 4).
